# An Empirical Assessment of Exposure Measurement Error and Effect Attenuation in Bipollutant Epidemiologic Models

**DOI:** 10.1289/ehp.1307772

**Published:** 2014-07-08

**Authors:** Kathie L. Dionisio, Lisa K. Baxter, Howard H. Chang

**Affiliations:** 1 National Exposure Research Laboratory, U.S. Environmental Protection Agency, Research Triangle Park, North Carolina, USA; 2 Department of Biostatistics and Bioinformatics, Emory University, Atlanta, Georgia, USA

## Abstract

Background: Using multipollutant models to understand combined health effects of exposure to multiple pollutants is becoming more common. However, complex relationships between pollutants and differing degrees of exposure error across pollutants can make health effect estimates from multipollutant models difficult to interpret.

Objectives: We aimed to quantify relationships between multiple pollutants and their associated exposure errors across metrics of exposure and to use empirical values to evaluate potential attenuation of coefficients in epidemiologic models.

Methods: We used three daily exposure metrics (central-site measurements, air quality model estimates, and population exposure model estimates) for 193 ZIP codes in the Atlanta, Georgia, metropolitan area from 1999 through 2002 for PM_2.5_ and its components (EC and SO_4_), as well as O_3_, CO, and NO_x_, to construct three types of exposure error: δ_spatial_ (comparing air quality model estimates to central-site measurements), δ_population_ (comparing population exposure model estimates to air quality model estimates), and δ_total_ (comparing population exposure model estimates to central-site measurements). We compared exposure metrics and exposure errors within and across pollutants and derived attenuation factors (ratio of observed to true coefficient for pollutant of interest) for single- and bipollutant model coefficients.

Results: Pollutant concentrations and their exposure errors were moderately to highly correlated (typically, > 0.5), especially for CO, NO_x_, and EC (i.e., “local” pollutants); correlations differed across exposure metrics and types of exposure error. Spatial variability was evident, with variance of exposure error for local pollutants ranging from 0.25 to 0.83 for δ_spatial_ and δ_total_. The attenuation of model coefficients in single- and bipollutant epidemiologic models relative to the true value differed across types of exposure error, pollutants, and space.

Conclusions: Under a classical exposure-error framework, attenuation may be substantial for local pollutants as a result of δ_spatial_ and δ_total_ with true coefficients reduced by a factor typically < 0.6 (results varied for δ_population_ and regional pollutants).

Citation: Dionisio KL, Baxter LK, Chang HH. 2014. An empirical assessment of exposure measurement error and effect attenuation in bipollutant epidemiologic models. Environ Health Perspect 122:1216–1224; http://dx.doi.org/10.1289/ehp.1307772

## Introduction

Most epidemiologic studies of the health effects of ambient air pollution have focused on adverse effects associated with single pollutants. In reality, humans are simultaneously exposed to a complex mixture of pollutants that can vary both spatially and temporally (Dominici et al. 2010). Epidemiologic analyses that have examined multipollutant health effects have typically relied on ambient monitoring data to estimate exposures (Hoffmann et al. 2012; Tolbert et al. 2007). Measurements from federal or state ambient monitoring networks often lack spatial and temporal coverage (Goldman et al. 2010; Sarnat et al. 2010) and do not account for exposures in different microenvironments (e.g., in-vehicle and in-home exposures) where infiltration (Sarnat et al. 2006; Weisel et al. 2005) and indoor sources (Baxter et al. 2007; Meng et al. 2009) can contribute substantially. There is, therefore, a potential for exposure measurement error that can lead to effect attenuation and reduced statistical power when measurements from ambient monitors are used as the exposure estimate in an epidemiologic study.

Complex relationships may exist between exposures to various pollutants, and between the exposure error associated with each pollutant. The magnitude of the exposure error may differ across pollutants (Tolbert et al. 2007). For example, pollutants with primarily local sources [e.g., carbon monoxide (CO), nitrogen oxides (NO_x_), elemental carbon (EC)] exhibit significant spatial heterogeneity (Goldman et al. 2010; Sarnat et al. 2010; Strickland et al. 2013) that may not be captured by central-site (CS) ambient monitors. Exposures estimated from ambient monitors for these pollutants may be associated with more error than monitor-based estimates for pollutants that are more spatially homogeneous [e.g., fine particulate matter (PM_2.5_; ≤ 2.5 μm in aerodynamic diameter), sulfate (SO_4_), ozone (O_3_)]. When exposure estimates do not take into account exposure factors such as time–location–activity patterns (including time spent indoors) (Monn 2001; Setton et al. 2011), significant indoor sources [e.g., gas stoves contributing to nitrogen dioxide (NO_2_) exposures] (Williams et al. 2012), or housing characteristics [e.g., air exchange rate (AER), pollutant infiltration] (Sarnat JA et al. 2013), exposure error may be greater.

Previous studies have predominantly focused on quantifying and accounting for exposure error in single-pollutant models (Sarnat et al. 2010; Setton et al. 2011; Strickland et al. 2013). Zeka and Schwartz (2004) focused on a method for the analysis of health effects in multipollutant studies that is resistant to measurement error. Among other findings, Zeka and Schwartz (2004) found an association between CO and daily mortality when traditional analysis did not, suggesting that a high degree of measurement error due to spatial heterogeneity of CO concentrations may be contributing to the difference in findings. In another study, Schwartz and Coull (2003) provided alternative methods for estimating the effect of two exposures on an outcome that reduced bias at the cost of a small-to-moderate reduction in power.

The objective of the present analysis was to examine exposure errors for multiple pollutants and provide insights on the potential for bias and attenuation of effect estimates in single- and bipollutant epidemiologic models. We used this approach to examine the robustness of the association for a pollutant of interest when a second pollutant is controlled for, that is, to examine the attenuation due to measurement errors present in both pollutants. In a previous analysis, alternative exposure estimates for ambient-generated PM_2.5_, EC, SO_4_, CO, NO_x_, and O_3_ were developed, and spatiotemporal patterns for each estimate were characterized in comparison with CS monitor measurements (Dionisio et al. 2013). The exposure estimates were used in an epidemiologic study in the Atlanta, Georgia, metropolitan area, using a time-series design to examine the association between daily exposure to ambient air pollution and daily emergency department (ED) visits for asthma/wheeze during a 4-year study period (1999–2002) (Sarnat SE et al. 2013). Using a modified set of the previously generated exposure estimates, we examined the exposure error and between-pollutant relationships and quantified potential attenuation of model coefficients in single- and bipollutant models at the ZIP code level for ambient-generated PM_2.5_, EC, SO_4_, CO, NO_x_, and O_3_ in Atlanta.

## Methods

*Estimates of exposure*. Three estimates of daily exposure to ambient PM_2.5_, EC, SO_4_, CO, NO_x_, and O_3_ were derived for 193 ZIP codes in the 20-county Atlanta metropolitan area for use in an epidemiologic analysis of cardiovascular and respiratory outcomes based on data from ED visits. Each metric builds on previous metrics, incorporating the coarser measurements and model estimates and becoming increasingly more finely resolved. The three estimation approaches, or “metrics,” for exposure to ambient pollution include *a*) CS: CS measurements; *b*) AQ: a hybrid of a statistical model for regional background and a dispersion model for the local contribution to ambient air quality; and *c*) PE: a stochastic population exposure model. We used the AERMOD (American Meteorological Society/Environmental Protection Agency Regulatory Model) dispersion model, version 09292, for the local contribution to the AQ metric, and the U.S. Environmental Protection Agency’s (EPA) Stochastic Human Exposure and Dose Simulation (SHEDS) model (Burke et al. 2001) for the PE metric. The contribution from indoor sources was not included in any of the approaches because of the desire to associate exposure to ambient pollution with the health outcome. All three approaches estimate exposures to ambient pollution at each ZIP code centroid in the study area. Daily estimates (8-hr maximum for O_3_, 24-hr average for other pollutants) for 1999–2002 were generated for the three exposure estimation approaches.

*CS measurements*. CS measurements for each pollutant were obtained from the Southeastern Aerosol Research and Characterization (SEARCH) network (http://www.atmospheric-research.com/studies/SEARCH/), the Assessment of Spatial Aerosol Composition in Atlanta (ASACA) network (Butler et al. 2003), and the U.S. EPA’s Air Quality System (AQS) monitoring network (http://www.epa.gov/ttn/airs/airsaqs/aqsweb/)(see Supplemental Material, Figure S1). Details regarding measurement methods, imputations for filling in missing data, and previous work using these monitors to characterize background air pollution levels have been reported previously (Dionisio et al. 2013; Metzger et al. 2004; Tolbert et al. 2000). Daily 24-hr average concentrations of PM_2.5_, EC, and SO_4_ were taken directly from monitor measurements. Hourly concentrations for CO and NO_x_ were aggregated to 24-hr averages, and hourly concentrations for O_3_ were aggregated to daily 8-hr maximum concentrations.

*AQ model estimates*. AQ model estimates were obtained by combining local- and regional-scale model results (based on CS measurements) to account for all major atmospheric processes, including local contributions (driven by local-scale variation in pollutant emissions and meteorology) and regional contributions (background levels associated with large-scale synoptic patterns). The sum of the modeled regional background contribution and the local contribution was computed hourly to obtain total modeled ambient air concentrations at each ZIP code centroid for each pollutant being studied. To obtain estimates of the regional background contribution, we modified an approach developed to provide population-weighted daily averages of ambient pollution concentrations (Ivy et al. 2008) to provide spatially resolved hourly estimates of regional background pollution by removing local-source impacts modeled by hour of day and day of week. Local-scale pollutant contributions for PM_2.5_, EC, SO_4_, CO, and NO_x_ at each ZIP code centroid were modeled using the AERMOD dispersion model, version 09292 (Cimorelli et al. 2005), which simulates concentrations of pollutants directly emitted into the atmosphere. Because O_3_ is formed by photochemical processes and has no direct emissions, O_3_ concentrations were not modeled with AERMOD. Similarly, the SO_4_ concentrations estimated from AERMOD were from direct vehicle exhaust emissions and did not include the secondary SO_4_ contribution due to photochemical transformations in the atmosphere. Further details on methodology and modeling of the regional contribution, local-scale contribution, and computation of the AQ metric estimates have been reported previously (Dionisio et al. 2013).

*PE model estimates*. We used the SHEDS model (Burke et al. 2001) to derive PE model estimates of daily population exposures to ambient pollution at each ZIP code centroid. The SHEDS model is a stochastic population exposure model that uses a probabilistic approach to estimate personal exposures for simulated individuals of a defined population based on ambient concentrations, distributions of residential AERs and particle infiltration parameters (i.e., penetration factors and deposition rates), and time spent in various microenvironments (e.g., home, office, school, vehicle) from a large database of human activity diaries. Key inputs to the model are the AQ metric estimates described above, time–location–activity data from the U.S. EPA’s Consolidated Human Activity Database (McCurdy et al. 2000), spatially varying local AERs (Sarnat JA et al. 2013), and census tract–level home-to-work commuting data (Bureau of Transportation Statistics 2000; U.S. EPA 2012). Penetration and decay parameters used in the model are specific to each pollutant; however, they do not vary spatially or temporally (see Supplemental Material, Tables S1 and S2). To derive model estimates for exposures to ambient pollution, consistent with the CS and AQ metrics, we excluded contributions from indoor source emissions for this analysis. For additional details, see Supplemental Material, “Population exposure metric.”

*Statistical analyses*. We computed ZIP code–level summary statistics for each exposure metric for each pollutant. Statistics included the annual mean normalized pollutant concentrations, as well as the variance across days of the normalized pollutant concentrations, for each exposure metric. To allow for comparisons across pollutants, we normalized ZIP code–specific pollutant concentrations for each exposure metric by dividing the daily pollutant concentration by the annual average CS measurement for that pollutant. We then compared the magnitude and spatial variability of normalized pollutant concentrations across pollutants and exposure metrics.

One standard approach for examining the health effects of multiple pollutants is to include each pollutant as an independent risk factor simultaneously in a single epidemiologic model (Bell et al. 2007; Tolbert et al. 2007). The correlation between the exposure estimates, the degree of exposure error for each pollutant, and the correlation of exposure errors between pollutants must all be considered in order to assess the impacts of exposure error on health risk estimates in a multipollutant model (Zeger et al. 2000; Zidek et al. 1996).

In the present analysis, exposure error, δ, was calculated as the difference between two exposure metrics. We present three types of exposure error (δ_spatial_, δ_population_, and δ_total_). The exposure error due to a lack of spatial refinement in the exposure estimate is represented by δ_spatial_ = *AQ* – *CS* because our air quality models add spatial variability to the AQ metric compared with CS measurements, which lack spatial variability because the same CS measurement was used to represent exposure in each ZIP code. Exposure error introduced when human exposure factors are not included in an exposure estimate is represented by δ_population_ = *PE* – *AQ*. Our PE metric includes variability due to human exposure factors such as time–location–activity patterns of individuals, commuting patterns, and infiltration of ambient pollutants to the indoor environment. A third type of exposure error, δ_total_ = *PE* – *CS*, represents the combined exposure error when both spatial variability and human exposure factors are not accounted for. δ_Total_ does not represent all potential sources of exposure error that may be present in a study; instead it represents the total exposure error that we were able to assess in this analysis. As with the pollutant concentrations, daily ZIP code–specific estimates of exposure error were normalized by dividing by the annual average CS measurement for that pollutant to allow for comparison across pollutants and types of exposure error. We also present the variance calculated across days of the normalized exposure error to aid in estimating the degree of bias and attenuation of model coefficients.

We calculated the between-pollutant Pearson correlations over time for each exposure estimation approach—and for each type of exposure error—to provide information on the collinearity of exposure estimates and exposure error that must be accounted for in a multipollutant model. Correlations were calculated for each ZIP code individually, allowing the range of correlations to be compared across the study domain.

Estimates of the level of attenuation of model coefficients for single- and bipollutant models are presented to aid in the interpretation of future epidemiologic models including two or more pollutants. The attenuation factor (λ) for a classical error, single-pollutant framework is calculated as

λ = 1/{1 + [var(δ)/var(*x*_fine_)]} [1]

β_observed_ = λ *×* β_true_, [2]

where δ is the exposure error, *x*_fine_ is the exposure metric with the greater degree of refinement (i.e., increased spatial resolution or inclusion of weighting by population factors), var(*x*_fine_) is the variance across days of *x*_fine_, and β represents the model coefficients. Assuming that the related epidemiologic analysis fits a time-series model separately for each ZIP code, β represents the association between the health outcome and the daily pollutant exposure. For simplicity, we present the attenuation factor λ*_x_*__1__ for pollutant *x*_1_ in a bipollutant model, assuming that pollutant *x*_2_ has no effect (β*_x_*__2__ = 0), given by the diagonal elements of

λ*_x_*__1__ = S(S + V)^–1^ [3]

β_observed,_*_x_*__1__ = λ*_x_*__1__ *×* β_true_*_,x_*__1__, [4]

where S is the covariance of the exposure metrics with the greater degree of refinement for *x*_1_ and *x*_2_, and V is the covariance of the exposure errors for *x*_1_ and *x*_2_. For the single- and bipollutant models, an attenuation factor of λ = 1 indicates no attenuation (i.e., β_observed_ = β_true_), and λ = 0 (i.e., β_observed_ = 0) indicates null results. An attenuation factor of λ > 1 indicates bias away from the null, and λ < 0 indicates that the estimated coefficient will be in the opposite direction of the true effect. For example, the λ associated with δ_spatial_ in a single-pollutant model reflects the attenuation of model coefficients due to error from incomplete characterization of the spatial variation in the concentration of the pollutant in question.

All statistical analyses were completed in R, version 2.15.1 (R Foundation for Statistical Computing; http://www.r-project.org/). All mapping was done in ArcGIS 10 (ESRI; http://www.esri.com/software/arcgis/).

## Results

This study builds on previous work in which single-pollutant epidemiologic models were used to estimate the association between daily counts of ZIP code–level ED visits and ZIP code–specific exposures using the three metrics (Sarnat JA et al. 2013; Sarnat SE et al. 2013). Related analyses also showed that the temporal variation in the AQ measure was not always more variable than temporal variation in the CS metric (Dionisio et al. 2013). The goal of the present analysis was to examine exposure error and between-pollutant relationships and how these differ by pollutant pair and exposure metric. Using the empirical covariance structures allowed us to assess potential attenuation of model coefficients in bipollutant epidemiologic models.

**Figure 1:**
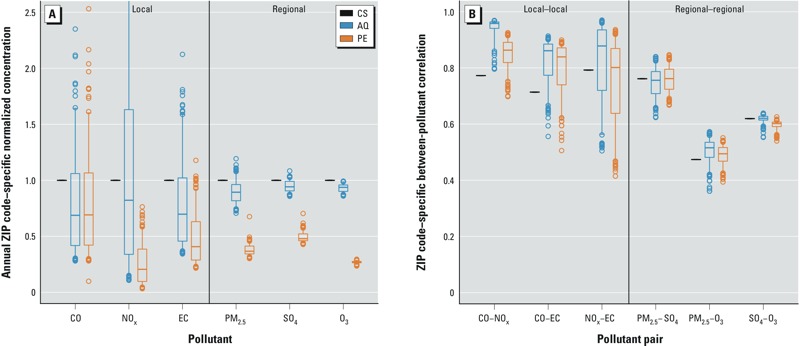
Relationships between the three ZIP code–specific exposure metrics. (*A*) Normalized exposure metrics for local and regional pollutants (see Supplemental Material, Figure S2A, for full extent of data and outliers). (*B*) Between-pollutant correlations of exposure for local–local and regional–regional pollutant pairs (see Supplemental Material, Figure S2B, for local–regional pollutant pairs). The bottoms and tops of boxes represent 25th and 75th percentiles, lines within boxes indicate the median, and the whiskers represent 5th and 95th percentiles; *n* = 193.

*Summary statistics for exposure metrics*. Figure 1A presents ZIP code–specific normalized exposure metrics averaged across the entire study period (see Supplemental Material, Figure S2A, for an expanded version that shows the full distributions for each metric). Distributions of pollutant concentrations differ by exposure metric—with the PE estimates being consistently equal to AQ estimates for CO or lower than AQ estimates for NO_x_, EC, PM_2.5_, SO_4_, and O_3_—due to the penetration and decay parameters used in the SHEDS model (see Supplemental Material, Tables S1 and S2). There was no spatial variability for the CS metric because the same CS measurement was used for all ZIP codes. However, when AQ or PE modeling was used, we observed considerable spatial variability [i.e., variation among the 193 ZIP code–specific estimates, as indicated in the box plots by a larger interquartile range (IQR), and a larger range from the 5th to 95th percentiles]. For all pollutants except CO, PE estimates exhibited a lower degree of spatial variability than AQ estimates. Local pollutants (CO, NO_x_, and EC) had relatively more spatial variability in their AQ and PE metrics than did regional pollutants (PM_2.5_, SO_4_, and O_3_), which was expected given the variation of local source emissions such as traffic at the ZIP code level.

*Between-pollutant correlations of exposure metrics*. Box plots of pairwise Pearson correlation coefficients of daily, ZIP code–specific exposure metrics for local–local and regional–regional pollutant pairs are presented in Figure 1B. All local–local and regional–regional pollutant pairs showed moderate-to-strong positive correlations for each metric; however, correlations for regional–regional pollutant pairs tended to be lower. For the regional–regional pollutant pairs, the median correlation for each pair was consistent across the three exposure metrics. In contrast, for each local–local pollutant pair, the correlation coefficient for CS measurements was lower than the median correlation for the AQ and PE metrics. Correlations of local–regional pollutant pairs were more varied and typically weaker than local–local and regional–regional pollutant pair correlations, with the exception of correlations of CO, NO_x_, and EC with PM_2.5_ (see Supplemental Material, Figure S2B).

Spatial variability (described by the width of the box plot) was present to varying degrees for correlations within the AQ and PE metrics, with more spatial variability present for local–local pollutant correlations than for regional–regional pollutant correlations, especially for the CO–EC and NO_x_–EC pairs (Figure 1B). The degree of spatial variability for regional–regional pollutant pairs was similar for both the AQ and PE metrics. There was no spatial variability present for the between-pollutant correlations of exposure for the CS metric, given that the same CS measurement was used for each ZIP code.

**Figure 2:**
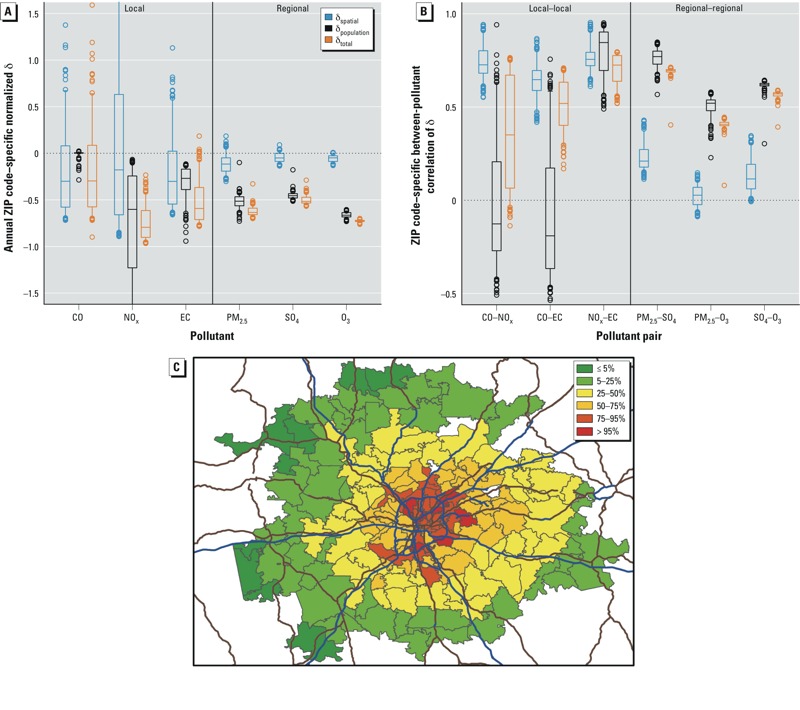
Relationships between the three types of exposure error. (*A*) Normalized exposure error for local and regional pollutants (see Supplemental Material, Figure S3A, for full extent of data and outliers). (*B*) Between-pollutant correlations of exposure error for local–local and regional–regional pollutant pairs (see Supplemental Material, Figure S3B, for local–regional pollutant pairs). The bottoms and tops of boxes represent 25th and 75th percentiles, lines within boxes indicate the median, and the whiskers represent 5th and 95th percentiles; *n* = 193. δ_spatial_ = AQ – CS; δ_population_ = PE – AQ; and δ_total_ = PE – CS. (*C*) Map showing δ_spatial_ of NO_x_ for ZIP codes in Atlanta metropolitan area. Blue and brown lines indicate major roads; colors indicate the percentile, where 5% = –0.85; 25% = –0.66; 50% = –0.18; 75% = 0.63; and 95% = 1.73.

*Summary statistics for exposure error*. The magnitude and spatial variability of the three types of normalized exposure error (δ_spatial_, δ_population_, and δ_total_) across pollutants are presented in Figure 2A (see Supplemental Material, Figure S3A, for the full distribution). The distribution of exposure error across ZIP codes was mostly negative (indicating that the exposure metric with a greater degree of refinement had a lower magnitude), although exposure errors were positive for a small number of ZIP codes. The magnitude of the exposure error varied by type of error, with the absolute value of exposure error greater for δ_population_ and δ_total_ than for δ_spatial_ for regional pollutants, and with mixed results for local pollutants. With δ_spatial_ near zero for the regional pollutants (the median absolute value of δ_spatial_ across ZIP codes was < 0.12, indicating similar magnitude for CS measurements and AQ estimates), their total exposure error (δ_total_) consisted mostly of exposure error due to human exposure factors (δ_population_), indicating greater differences in magnitude for AQ estimates relative to PE estimates.

To assess the potential for spatially differential exposure error, we compared the spatial variability of exposure errors across ZIP codes. With the exception of δ_population_ for CO, the spatial variability of exposure error was greater for local pollutants than regional pollutants (Figure 2A,C). For local pollutants, spatial variability was present to varying degrees across all types of error [smallest range of 5th to 95th percentiles of normalized exposure error, –0.64 to –0.13 (EC, δ_population_); largest range, –0.85 to 1.73 (NO_x_, δ_spatial_)], with the exception of δ_population_ for CO, which was near zero because of the use of a penetration factor of 1 (i.e., assuming free flow of outdoor and indoor air) in the SHEDS model for CO (see Supplemental Material, Table S2). In contrast, regional pollutants exhibited little spatial variability across types of exposure error, and the degree of spatial variability was consistent within a pollutant and across types of error.

*Between-pollutant correlations of exposure error*. The collinearity of exposure error was examined based on Pearson correlations between daily exposure error for local–local and regional–regional pollutant pairs (Figure 2B; see also Supplemental Material, Figure S3B, for local–regional pairs). The correlation of exposure error was highly dependent on both pollutant pair and type of exposure error. Between-pollutant correlations of exposure error were mostly positive, although there were some ZIP codes with negative correlations, especially for CO. The correlation of exposure error due to a lack of spatial refinement (δ_spatial_) was moderate to strong for local–local pollutant pairs (median correlation over all ZIP codes ranged from 0.65 to 0.76), and relatively weak for regional–regional pollutant pairs (median correlation ranged from 0.03 to 0.21). The correlation for δ_population_ showed a near opposite trend, with weak, negative correlations of δ_population_ for CO–NO_x_ and CO–EC (–0.13 and –0.19, respectively), and moderate-to-strong positive correlations of δ_population_ for NO_x_–EC (0.85) and the regional–regional pollutant pairs (ranged from 0.52 to 0.77). The magnitude of the correlation of total exposure error (δ_total_) between local–local and regional–regional pollutant pairs varied, with median correlations of δ_total_ across ZIP codes ranging from 0.35 to 0.72 (Table 1, Figure 2B).

**Table 1:** Parameters affecting attenuation and bias in bivariate pollutant models of pairs of local pollutants (CO, NO_x_, and EC) or pairs of regional pollutants (PM_2.5_, SO_4_, and O_3_).

Parameter	CO–NO_x_^*a*^	CO–EC	NO_x_–EC	PM_2.5_–SO_4_	PM_2.5_–O_3_	SO_4_–O_3_
AQ Corr(*x*_1_,*x*_2_)	0.96	0.86	0.88	0.76	0.52	0.62
PE Corr(*x*_1_,*x*_2_)	0.86	0.84	0.80	0.76	0.49	0.60
δ_spatial_
Var(δ_1_)^*b*^	0.25	0.25	0.83	0.04	0.04	0.05
Var(δ_2_)^*b*^	0.83	0.30	0.30	0.05	0.02	0.02
Corr(δ_1_, δ_2_)	0.73	0.65	0.76	0.21	0.03	0.11
δ_population_
Var(δ_1_)	0.00	0.00	0.32	0.09	0.09	0.10
Var(δ_2_)	0.32	0.05	0.05	0.10	0.11	0.11
Corr(δ_1_, δ_2_)	–0.13	0.85	0.85	0.77	0.52	0.62
δ_total_
Var(δ_1_)	0.25	0.80	0.80	0.12	0.12	0.16
Var(δ_2_)	0.80	0.33	0.33	0.16	0.16	0.16
Corr(δ_1_, δ_2_)	0.35	0.72	0.72	0.70	0.41	0.57
Corr, correlation. Data are presented as medians across all ZIP codes. ^***a***^The first pollutant in each pair corresponds to *x*_1_ and the second to *x*_2_. ^***b***^Var(δ) represents variance of normalized exposure error.						

Local–local and regional–regional pollutant pairs showed a moderate degree of spatial variability in the correlation of δ_spatial_ (Figure 2B). The patterns of spatial variability of the correlation of δ_population_ are more varied, with local–local pollutant pairs showing a larger degree of spatial variability than regional–regional pollutant pairs (5th to 95th percentile for correlation coefficients of 0.56 to 0.93 for NO_x_–EC, –0.42 to 0.63 for CO–NO_x_, and –0.46 to 0.59 for CO–EC). Although there was a large range of correlations across ZIP codes for δ_population_ for CO–NO_x_ and CO–EC in particular, the bulk of the correlations across the study area were relatively weak (25th to 75th percentile for correlation coefficients of –0.27 to 0.21 for CO–NO_x_ and –0.37 to 0.17 for CO–EC. As reflected in comparisons of δ_spatial_ and δ_population_, we saw greater spatial variability in the correlation of δ_total_ for the local–local pollutant pairs, and very little spatial variability in the correlation of δ_total_ for the regional–regional pairs.

**Figure 3:**
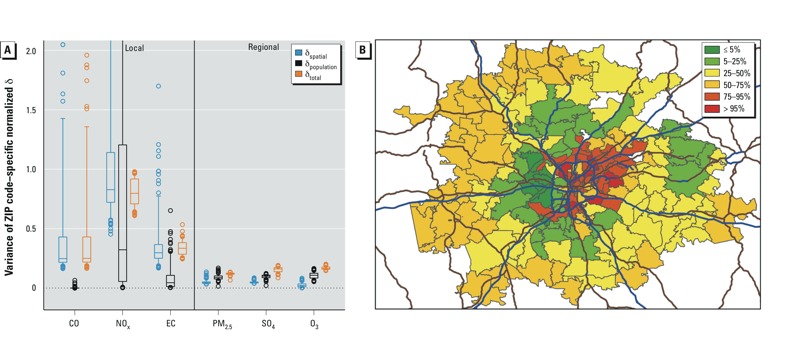
Variance of exposure error. (*A*) Variance of normalized exposure error (δ) for local and regional pollutants (see Supplemental Material, Figure S4, for full extent of data and outliers). The bottoms and tops of boxes represent 25th and 75th percentiles, lines within boxes indicate the median, and the whiskers represent 5th and 95th percentiles; *n* = 193. δ_spatial_ = AQ – CS; δ_population_ = PE – AQ; and δ_total_ = PE – CS. (*B*) Map showing variance of δ_spatial_ of NO_x_ for ZIP codes in the Atlanta metropolitan area. Blue and brown lines indicate major roads; colors represent percentiles, where 5% = 0.58; 25% = 0.72; 50% = 0.83; 75% = 1.14; and 95% = 4.14.

*Variance of exposure error*. For regional pollutants (PM_2.5,_ SO_4_, and O_3_), variance across days of the normalized exposure error had very little spatial variability (i.e., box plots of the variance of normalized exposure error are narrow) and was < 0.20 for any type of error in any ZIP code (Figure 3A). In comparison, with the exception of δ_population_ for CO, variance of the exposure error, as well as spatial variability of the variance, was present for local pollutants (Figure 3; see also Supplemental Material, Figure S4, for the full distribution). For the local pollutants, the magnitude and spatial variability of the variance of normalized error differed depending on pollutant and type of error, with the variance of δ_spatial_ and δ_population_ for NO_x_ having the largest range of spatial variability, whereas the variance of exposure error for EC exhibited more modest spatial variability.

*Attenuation of model coefficients*. By compiling empirically determined parameters related to the between-pollutant relationships and their associated exposure error (Table 1), and utilizing Equation 3, we were able to quantify the potential attenuation of model coefficients in a bipollutant model. Table 1 presents the median values across all ZIP codes of the correlations over time and the variances across days for pollutant concentrations and their associated exposure errors. To calculate the attenuation factors, we used the individual ZIP code–specific values of these parameters (Figures 1B, 2B, and 3A; for the full range of parameter values across all ZIP codes, see Supplemental Material, Figures S2A, S3B, and S4).

**Figure 4:**
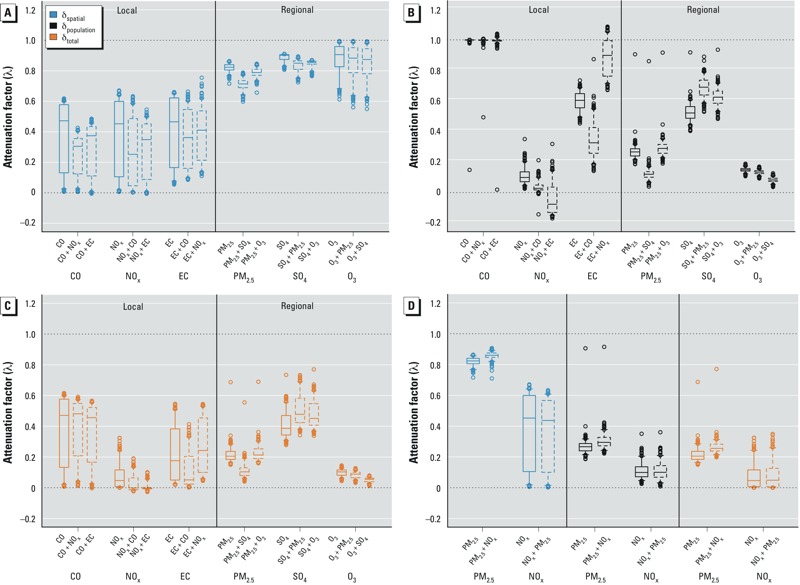
Attenuation of model coefficients in a classical-error, single-pollutant framework and in bipollutant models, assuming that one pollutant has an effect and one pollutant has no effect. (*A*) λ for local–local and regional–regional pollutant pairs due to δ_spatial_. (*B*) λ local–local and regional–regional pollutant pairs due to δ_population_. (*C*) λ for local–local and regional–regional pollutant pairs due to δ_total_. (*D*) λ due to δ_spatial_, δ_population_, and δ_total_ for a local–regional pollutant example (i.e., PM_2.5_, PM_2.5_ and No_x_). See Supplemental Material, Figure S5, for all local–regional pollutant pairs. The bottoms and tops of boxes represent 25th and 75th percentiles, lines within boxes indicate the median, and the whiskers represent 5th and 95th percentiles; *n* = 193. Solid-line boxes indicate attenuation factors for single-pollutant models, and dashed-line boxes represent attenuation factors for bipollutant models. The top row of *x*-axis labels indicates the pollutant effect being considered (e.g., CO), and the bottom row of *x*-axis labels indicates the relevant model and the presence or absence of co-pollutants (e.g., CO, CO + NO_x_, CO + EC). For example, for the three plots representing attenuation factors for CO, the first plot is the set of attenuation factors for CO in a single-pollutant model; the second is the attenuation factors for CO in a bipollutant model with NO_x_, assuming that NO_x_ has no effect; and the ^third^ is the attenuation factors for CO in a bipollutant model with EC, assuming that EC has no effect. λ = 1 indicates no attenuation, λ = 0 indicates null results.

Figure 4 presents the potential attenuation factors for single- and bipollutant epidemiologic models, based on empirical estimates of the relationships between exposure metrics and their exposure error. The attenuation factors presented for bipollutant models were based on the assumption that one pollutant has a true effect on the health outcome and the other pollutant has no effect. For δ_spatial_, we saw a clear distinction between local and regional pollutants, with more attenuation (typically, λ < 0.6) for both single- and bipollutant models of local pollutants, and less attenuation for regional pollutants (typically, λ > 0.6) [Figure 4A; λ = 1 indicates no attenuation (i.e., β_observed_ = β_true_), λ = 0 indicates null results, λ > 1 indicates bias away from the null, and λ < 0 indicates that the estimated coefficient will be in the opposite direction of the true effect]. The addition of a co-pollutant appears to increase attenuation. Results for δ_population_ and δ_total_ are more varied, with attenuation factors depending on the pollutant and co-pollutant (Figures 4B,C). For δ_spatial_ and δ_total_, we observed notable spatial variability in the attenuation factors (evidenced by wider box plots) for local pollutants (except for δ_total_ for NO_x_). For δ_population_, and regional pollutants for δ_spatial_ and δ_total_, the degree of spatial variability depends on the type of exposure error, pollutant, and co-pollutants.

For comparison, the attenuation factors for a bipollutant model with one local (NO_x_) and one regional (PM_2.5_) pollutant are presented in Figure 4D, showing significant differences in the attenuation factor across types of exposure error but smaller differences between single- and bipollutant models. See Supplemental Material, Figure S5, for the attenuation factors for bipollutant models for all local–regional pollutant pairs. Results occasionally showed bias away from the null (λ > 1) for some bipollutant combinations because of the strong correlations in both pollutant concentrations and exposure errors.

## Discussion

An improved understanding of the degree of exposure error among pollutants and their dependent structure is needed to properly interpret results from epidemiologic models that include multiple pollutants. By examining three different exposure metrics and three types of associated exposure measurement errors, we were able to empirically estimate bipollutant relationships and the potential for attenuation of model coefficients in related bipollutant epidemiologic models. For bipollutant models with local–local pollutant pairs, δ_spatial_ and δ_total_ were likely to introduce attenuation of model coefficients given the high correlations between local pollutant concentrations [corr(*x*_1_, *x*_2_] > 0.80 for all local–local pollutant pairs), unequal and nonzero variance of the exposure error for each pollutant [0.25 < var(δ) < 0.83], and moderate-to-high correlation of the exposure error for each pollutant pair [corr(δ_1_, δ_2_) > 0.52, except CO–NO_x_ for δ_total_]. For regional–regional pollutant pairs, the attenuation of model coefficients was likely to be minimal given the relatively low variance of the exposure error [var(δ) < 0.16 for all regional pollutants and types of exposure error]. The empirical quantification of the above parameters resulted in a predicted attenuation factor due to δ_spatial_ that was typically < 0.6 for single- and bipollutant models of local pollutants, with less attenuation for regional pollutant models (typically, λ > 0.6) and more varied results for δ_population_ and δ_total_.

The mean over all ZIP codes of AQ metric estimates that incorporated both regional background and local pollution contributions are similar in magnitude to CS measurements, although AQ metric estimates can exhibit spatial variability depending on local traffic patterns within the ZIP code, particularly for local pollutants. With the exception of CO, PE metric estimates for each pollutant were lower than their corresponding CS measurement because of the infiltration and decay parameters incorporated into the SHEDS human exposure model and the inclusion of time–activity data based on diaries that indicated that individuals spent the majority of their time indoors. PE metric estimates for CO were similar to AQ metric estimates because the penetration parameter for CO was set to 1 (i.e., assuming full penetration of CO from the outdoor to the indoor environment). Pollutant contributions from indoor sources were not included in this study; thus, the PE metric represents indoor and outdoor exposures to ambient pollution originating outdoors only.

Air quality models introduce spatial variability into AQ exposure estimates that is not captured when a single CS measurement is used for all ZIP codes in a study area. Spatial variability was much greater for pollutants with predominantly local sources (CO, NO_x_, and EC) compared with pollutants dominated by regional source contributions (PM_2.5_, SO_4_, and O_3_). This increase in spatial variability for local pollutants was mainly due to differences in traffic volume and patterns among different ZIP codes. Between-pollutant correlations were strong for local–local pollutant pairs, and moderate to strong for regional–regional pairs, reflecting the common emissions sources contributing to pollutant concentrations within each pair.

As expected, total exposure error (δ_total_) for regional pollutants was made up mostly of exposure error due to human exposure factors (e.g., time–activity patterns, AER in the home), with a small contribution from unmeasured spatial variability. In contrast, for the local pollutants NO_x_ and EC, there were substantial exposure error contributions from both human exposure factors and spatial heterogeneity in ambient concentrations. For CO, we saw a near-zero contribution from δ_population_ (due to full penetration of CO indoors).

*Potential impact of attenuation on epidemiologic model coefficients*. In a multipollutant model, the absolute magnitude of this bias will depend on the variance of the exposure error, the correlation between exposure estimates, and the correlation between exposure errors.

The present analysis builds upon the hypothetical simulation presented by Zeger et al. (2000) of predicted bias in regression coefficients in a bipollutant epidemiologic model. In a bipollutant model, we may not be concerned with bias if two regional pollutants are included because of the near-zero (δ_spatial_) and very low (δ_population_ and δ_total_) variance of exposure error for regional pollutants (Figure 3A, Table 1). However, in a bipollutant model including two local pollutants, there is the potential for bias and attenuation of model coefficients because of a higher degree of variance of exposure error. The effect in bipollutant models that include one local and one regional pollutant will vary, depending on the pollutant pair. In addition, empirically determined attenuation factors for single- and bipollutant models show that the potential for attenuation in the estimated effects can be quite substantial for many pollutants and exposure error types, in particular for local pollutants (with the exception of δ_population_ for CO) (Figure 4).

In addition to the potential for bias, the results presented here show that spatial variability is present in the exposure error for local pollutants and in the between-pollutant correlations of exposure error for local–local pollutant pairs. Figure 3B shows how the variance of spatial exposure error for NO_x_ changed across the study domain, with the variance of spatial exposure error being highest in the urban core (within and immediately surrounding the blue circular line indicating a major road), lowest in the central ring of our study domain (ring surrounding the urban core), and increasing slightly again as you extend to the western boundary of the study domain. These results highlight the importance of characterizing intraurban variations in exposure to avoid spatially varying differential exposure error. This is a particular concern when examining effect modification of air pollution health risks obtained without spatially resolved exposure estimates. For example, observed effect modification by ZIP code–level socioeconomic measures (Sarnat SE et al. 2013), which exhibit strong spatial patterns, may be due at least in part to varying degrees of attenuation bias from spatially differential exposure error.

Finally, when multiple pollutants are included simultaneously in a model of associations with health outcomes, bias away from the null may also occur. “Effect transfer” (Zidek et al. 1996) occurs when two correlated pollutants are measured with differential exposure error, and the effect of the pollutant measured with more error is transferred to the pollutant measured with less error. In this case, a pollutant without an effect on an outcome may become associated with it.

*Limitations*. Limitations of this study include uncertainties in the more refined exposure metric estimates (including that small area variations in pollutant concentrations may not be resolved due to sparsely distributed measurements used as inputs) and the exclusion of the influence of indoor sources. Although it is commonplace to use exposure to ambient sources as a proxy for an individual’s total exposure in an epidemiologic study, the inclusion of indoor sources would further enhance study findings.

Our findings may be generally applicable to study areas with similar source contributions (e.g., predominantly traffic-related local sources) and housing characteristics (e.g., low AERs). For any study area, the methods and models presented here may be applied if appropriate input data sets are used. With the exception of the locally derived AERs, all Atlanta-specific input data sets (e.g., CS pollutant measurements, traffic patterns, local emissions) were extracted from larger, publically available databases maintained by federal and state agencies; thus, similar input data sets for any study area could be compiled. If local AERs were not available, estimates could be made based on published distributions of AERs from various parts of the country.

Although the magnitudes of effects may differ, we expect that general conclusions from our analysis will be applicable to other geographic areas. For example, most study regions will have some pollutant concentrations dominated by regional sources that are likely to remain spatially homogeneous and some pollutant concentrations dominated by local sources that are likely to be spatially heterogeneous within the study area. Thus, we believe that our conclusions about the spatial variability of exposure error being present, and the general likelihood of bias due to measurement error for certain pollutants, are likely to apply across studies.

In calculating the attenuation factor, we assumed a classical exposure measurement error framework. We recognize this is a strong assumption, but we feel it is more appropriate than assuming a Berkson error framework because the CS does not necessarily represent “average” exposure for any ZIP code on any given day. Because exposure measurement error is likely to contain both classical and Berkson type errors, depending on the pollutants and study design, the assumption of a solely classical error framework implies limited applicability. Moreover, an assumption of our assessment of attenuation was that the effect estimate is not subject to residual confounding, the association between pollutant concentration and the health outcome is linear, and there is no effect modification between the pollutant association in a bipollutant model. Further, we have implicitly assumed that the only bias present is additive (the present analysis does not consider multiplicative bias), and this should not impact the regression slope. Last, although empirical covariance structures and exposure errors have been used to quantify potential attenuation in bipollutant models (assuming only one pollutant has an effect on the health outcome), our analysis does not address the potential for effect transfer in a bipollutant model when both pollutants have an effect on the health outcome. A simulation study including the covariance structures of data presented here is warranted to quantify the effect on model coefficients in a multipollutant model.

In addition to the role of exposure error, additional factors must be considered as researchers further investigate epidemiologic analyses that include multiple pollutants. These include the possibility of nonlinear relationships of the various pollutants with the health outcome, interaction or synergism among pollutants included in a single epidemiologic model, and the possibility of the high correlation we have seen among pollutants leading to one pollutant appearing to be associated with the health outcome in an epidemiologic model when a correlated pollutant is the true causal association. A future simulation study that examines the applicability of the classical exposure measurement error framework and the degree of effect attenuation and transfer is warranted.

## Conclusions

This analysis is one of the first to quantify the effects of correlated exposure measurement error in bipollutant models (Chang et al. 2011). To our knowledge, this is the first study to look in detail at the effects of spatial variation using dispersion models and stochastic personal exposure simulators in a multipollutant context. We used empirical relationships to show the potential for bias (particularly effect attenuation) in epidemiologic model coefficients for bipollutant models [particularly for local pollutants (CO, NO_x_, and EC)] due to the presence of variance in the exposure error and correlation between pollutants and their errors. Further, we found evidence of the potential for spatially varying attenuation and bias due to the spatial variability present in these parameters on the ZIP code level. As researchers move toward multipollutant approaches, we must recognize the potential effects on model coefficients depending on the relationships that exist between pollutants and their errors.

## Supplemental Material

(952 KB) PDF
