# Supplementary material for: An Empirical Assessment of Exposure Measurement Error and Effect Attenuation in Bipollutant Epidemiologic Models
Source: Environ Health Perspect. 2014 Jul 8;122(11):1216–24. doi: 10.1289/ehp.1307772 (PMC4216163; doi:10.1289/ehp.1307772)
Supplement: (952 KB) PDF [file ehp.1307772.s001.508.pdf]

Environmental Health Perspectives doi:10.1289/ehp.1307772

Supplemental Material

An Empirical Assessment of Exposure Measurement Error and Effect Attenuation in Bipollutant Epidemiologic Models

Kathie L. Dionisio, Lisa K. Baxter, and Howard H. Chang

Table of Contents **Page**

Population exposure metric 2

Table S1. Input parameters for the SHEDS model: $PM_{2.5}$, SO_4, and EC 3

Table S2. Input parameters for the SHEDS model: O_3, CO, and NO_x 4

Figure S1. Map of metropolitan Atlanta with monitoring site locations and population density. 5

Figure S2. Relationships between three exposure metrics 6

Figure S3. Relationships between three types of measurement error 8

Figure S4. Variance of normalized exposure error: full extent of data and outliers (zoomed out version of Figure 3a 10

Figure S5. Attenuation of model coefficients in a classical error, single pollutant framework, and in bi-pollutant models, assuming one pollutant has an effect, and one pollutant has no effect 11

Population exposure metric

Ambient pollutant concentrations supplied at ZIP code centroids (as described in the CS metric section above), were used to represent ambient outdoor concentrations for calculation of exposure at the home, outside the home, and the home garage for groups of simulated individuals. Individuals were sampled by census tract as is normally done in SHEDS Air. The target was for 100 simulated individuals to be assigned to each of the 193 ZIP codes of interest. To obtain ZIP code level exposure estimates, individuals were also assigned to a ZIP code polygon. Because it is common for census tracts to overlay two or more ZIP codes, an area weighted scheme was used to define the likelihood of an individual in a specific census tract being assigned to a specific ZIP code.

To account for exposure during commuting and travel, each simulated individual's work location was selected stochastically based on probabilities derived from the 2000 U.S. Census Tract-toTract commuting data. Individuals can commute to any census tract overlapping the ZIP codes of interest. Workers commuting out of the sampling area (~3% of workers) are removed from the population and replaced with another worker that commutes within the sampling area. Travel other than work is limited to the closest 20 census tracts within the sampling area, and within 10 miles of an individual's home census tract. Each individual is assigned to 3 nearby census tracts to which they may travel for a variety of reasons (e.g. shopping, church, other facilities related to microenvironments in the CHAD database). Some facilities are always in the same census tract for an individual (e.g. church), and others are assigned randomly each trip (e.g. retail stores). Air quality during travel is based on the ZIP code associated with the origin of the travel.

Penetration and decay parameters can be found in Tables S1 and S2.

Table S1. Input parameters for the SHEDS model: $PM_{2.5}$, SO_4, and EC.

**Micro-environment** *Equation parameter or sub-environment*	**$PM_{2.5}$**	**SO_4**	**EC**
**Home Indoor**[a]			
Penetration, P (unitless)[b]	P_i = N[0.89, 0.06] P_d = N[P_i, 0.03*P_i] Limits = [0.76, 1]	P_i = N[0.95, 0.07] P_d = N[P_i, 0.03*P_i] Limits = [0.80, 1]	P_i = N[0.96, 0.04] P_d = N[P_i, 0.03*P_i] Limits = [0.87, 1]
Deposition rate, k (h^{-1})[c]	k_i = N[0.39, 0.09] k_d = N[k_i, 0.05*k_i] Limits = [0.19, 0.58]	k_i = N[0.19, 0.04] k_d = N[k_i, 0.05*k_i] Limits = [0.10, 0.28]	k_i = N[0.10, 0.02] k_d = N[k_i, 0.05*k_i] Limits = [0.06, 0.14]
**Indoors**[d,e,f]			
Attached garage	$C_{ambient}$	$C_{ambient}$	$C_{ambient}$
Office	Slope = 0.18 Intercept = 3.6 Residual = N[0, 2.9]	Scaling Factor = N[0.450, 0.382] Limits = [0, -]	Scaling Factor = N[0.390, 0.339] Limits = [0, -]
Store	Slope = 0.75 Intercept = 9 Residual = N[0, 2.1]	Scaling Factor = N[0.479, 0.223] Limits = [0, -]	Scaling Factor = N[0.606, 0.351] Limits = [0, -]
School	Slope = 0.6 Intercept = 6.8 Residual = N[0, 5.4]	Scaling Factor = N[0.511, 0.241] Limits = [0, -]	Scaling Factor = N[0.550, 0.152] Limits = [0, -]
Restaurant	Slope = 1.0 Intercept = 9.8 Residual = N[0, 10]	Scaling Factor = N[1.0, 0.457] Limits = [0, -]	Scaling Factor = N[0.367, 0.153] Limits = [0, -]
Parking garage	Same as Other	Same as Other	Same as Other
Service station	$C_{ambient}$	$C_{ambient}$	$C_{ambient}$
Other	Slope = 0.85 Intercept = 8.4 Residual = N[0, 4]	Scaling Factor = N[0.340, 0.391] Limits = [0, -]	Scaling Factor = N[0.590, 0.423] Limits = [0, -]
**Outdoors**[a,f]			
Home	$C_{ambient}$	$C_{ambient}$	$C_{ambient}$
Near street[e]	Scaling Factor = N[0.974, 0.185] Limits = [0.7, 1.3]	$C_{ambient}$	$C_{ambient}$
Other	$C_{ambient}$	$C_{ambient}$	$C_{ambient}$
**In-vehicle**[a,d,e]	Slope = 0.7125 Intercept = 0 Residual = N[0, 6.64] Limits = [-12, 20]	Scaling Factor = N[0.961, 0.129] Limits = [0, -]	Scaling Factor = N[1.633, 1.088] Limits = [0, -]

[a]N[x,y] = Normal[mean, standard deviation]. [b]P_i = individual penetration, P_d = daily penetration, with exposure concentrations calculated using mass-balance. [c]k_i = individual decay, k_d = daily decay, with exposure concentrations calculated using mass-balance. [d]When "slope" and "intercept" are provided, implemented as Slope * $C_{ambient}$ + Intercept + Residual using linear regression. [e]When "scaling factor" is provided, implemented as Scaling Factor * $C_{ambient}$, with Limits = [minimum, maximum], implemented using linear regression; see footnote 8 for definition of $C_{ambient}$. [f]$C_{ambient}$ = concentration of the pollutant in question, in the ambient (outdoor) air of the microenvironment under consideration.

Table S2. Input parameters for the SHEDS model: O_3, CO, and NO_x.

**Micro-environment**<br>*Equation parameter or sub-environment*	O_3	CO	NO_x
**Home Indoor**			
Penetration, P (unitless)[a]	$P_d = 1$	$P_d = 1$	$P_d = 1$
Deposition Rate, k (h^{-1})[b,c,d]	k_d = LN[2.51, 1.53]<br>Limits = [0.95, 8.05]	$k_d = 0$	k_d = U[1.02, 1.45]
**Indoors**[c,e,f,g,h]			
Attached garage	Same as home indoors	$C_{ambient}$	$C_{ambient}$
Office	$P_d = 1$<br>k_d = LN[2.51, 1.53]<br>Limits = [0.95, 8.05]<br>AER = LN[1.109, 3.015]<br>Limits = [0.07, 13.8]	$C_{ambient}$	$C_{ambient}$
Store	$P_d = 1$<br>k_d = LN[2.51, 1.53]<br>Limits = [0.95, 8.05]<br>AER = LN[1.109, 3.015]<br>Limits = [0.07, 13.8]	$C_{ambient}$	$C_{ambient}$
School	$P_d = 1$<br>k_d = LN[2.51, 1.53]<br>Limits = [0.95, 8.05]<br>AER = LN[1.109, 3.015]<br>Limits = [0.07, 13.8]	$C_{ambient}$	$C_{ambient}$
Restaurant	$P_d = 1$<br>k_d = LN[2.51, 1.53]<br>Limits = [0.95, 8.05]<br>AER = LN[1.109, 3.015]<br>Limits = [0.07, 13.8]	$C_{ambient}$	$C_{ambient}$
Parking garage	Scaling Factor = N[0.755, 0.203]<br>Limits = [0.422, 1.0]	$C_{ambient}$	$C_{ambient}$
Service station	$C_{ambient}$	$C_{ambient}$	$C_{ambient}$
Other	Same as office, store, school, restaurant	$C_{ambient}$	$C_{ambient}$
**Outdoors**[f,g,h]			
Home	$C_{ambient}$	$C_{ambient}$	$C_{ambient}$
Near street[e]	Scaling Factor = N[0.755, 0.203]<br>Limits = [0.422, 1.0]	$C_{ambient}$	$C_{ambient}$
Other	$C_{ambient}$	$C_{ambient}$	$C_{ambient}$
**In-vehicle**[d,f,g,h]	Scaling Factor 1 = N[0.755, 0.203]<br>Limits = [0.422, 1]<br>Scaling Factor 2 = N[0.3, 0.232]<br>Limits = [0.1, 1]	$C_{ambient}$	Scaling Factor = U[0.6, 1]

[a]P_i = individual penetration, P_d = daily penetration, with exposure concentrations calculated using mass-balance. [b]k_i = individual decay, k_d = daily decay, with exposure concentrations calculated using mass-balance. [c]LN[x,y] = Lognormal[geometric mean, geometric standard deviation]. [d]U[x,y] = Uniform[minimum, maximum]. [e]AER = air exchange rate. [f]N[x,y] = Normal[mean, standard deviation]. [g]When "scaling factor" is provided, implemented as Scaling Factor * $C_{ambient}$, with Limits = [minimum, maximum], implemented using linear regression; see footnote 8 for definition of $C_{ambient}$. [h]$C_{ambient}$ = concentration of the pollutant in question, in the ambient (outdoor) air of the microenvironment under consideration.

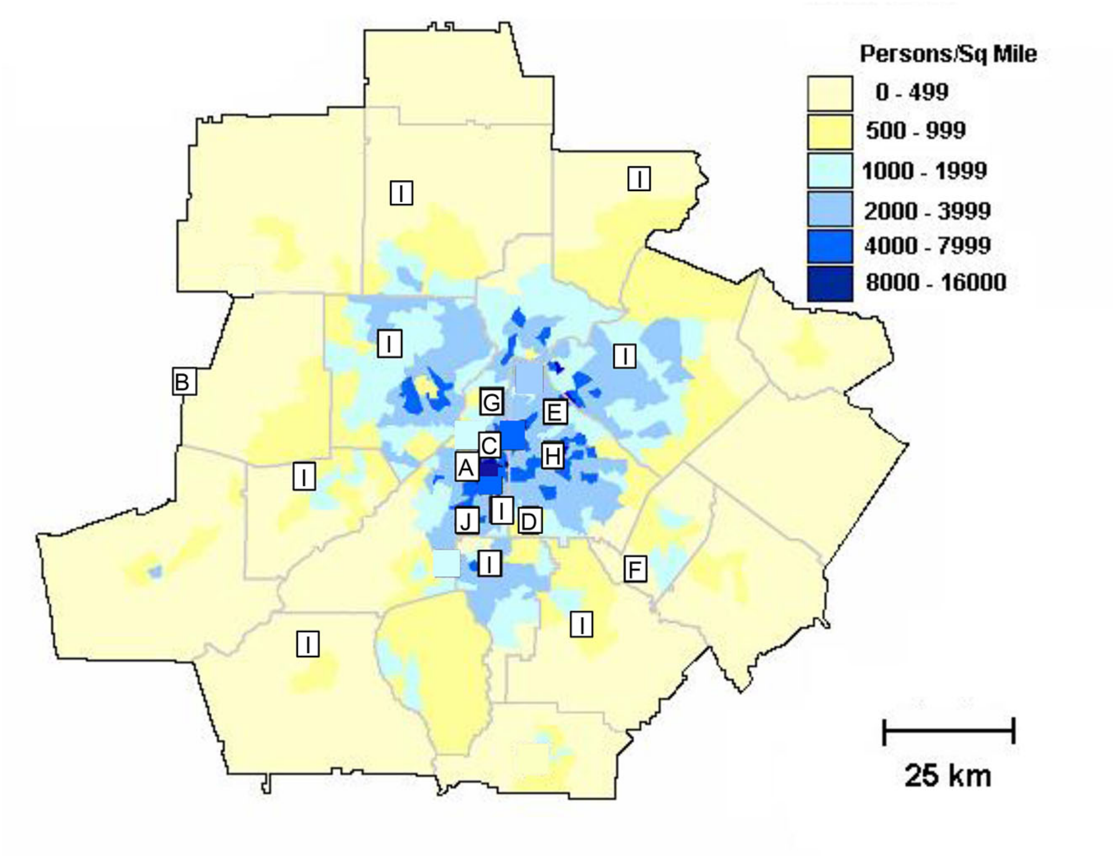

A	Jefferson Street, SEARCH (*1,2,3,4,5*)
B	Yorkville, SEARCH (*1,2,3,4,5*)
C	Georgia Tech, AQS (*1*)
D	South DeKalb, STN & ASACA (*1,3,4*)
E	Tucker, AQS & ASACA (*1,3,4*)
F	Conyers Monastery, AQS (*1,3*)
G	Roswell Road, AQS (*2*)
H	Dekalb Tech, AQS (*2*)
I	various ozone monitors, AQS (*3*)
J	Fort McPherson, ASACA (*4*)

Figure S1. Map of metropolitan Atlanta with monitoring site locations and population density. Letters reference monitor locations. The table identifies station name, network, and air pollutants monitored, with air pollutants indicated by numbers [1 = NO_2/NO_x, 2 = CO, 3 = O_3, 4 = $PM_{2.5}$ mass, 5 = $PM_{2.5}$ composition (SO_4, EC)]. Population density is from 2000 Census data.

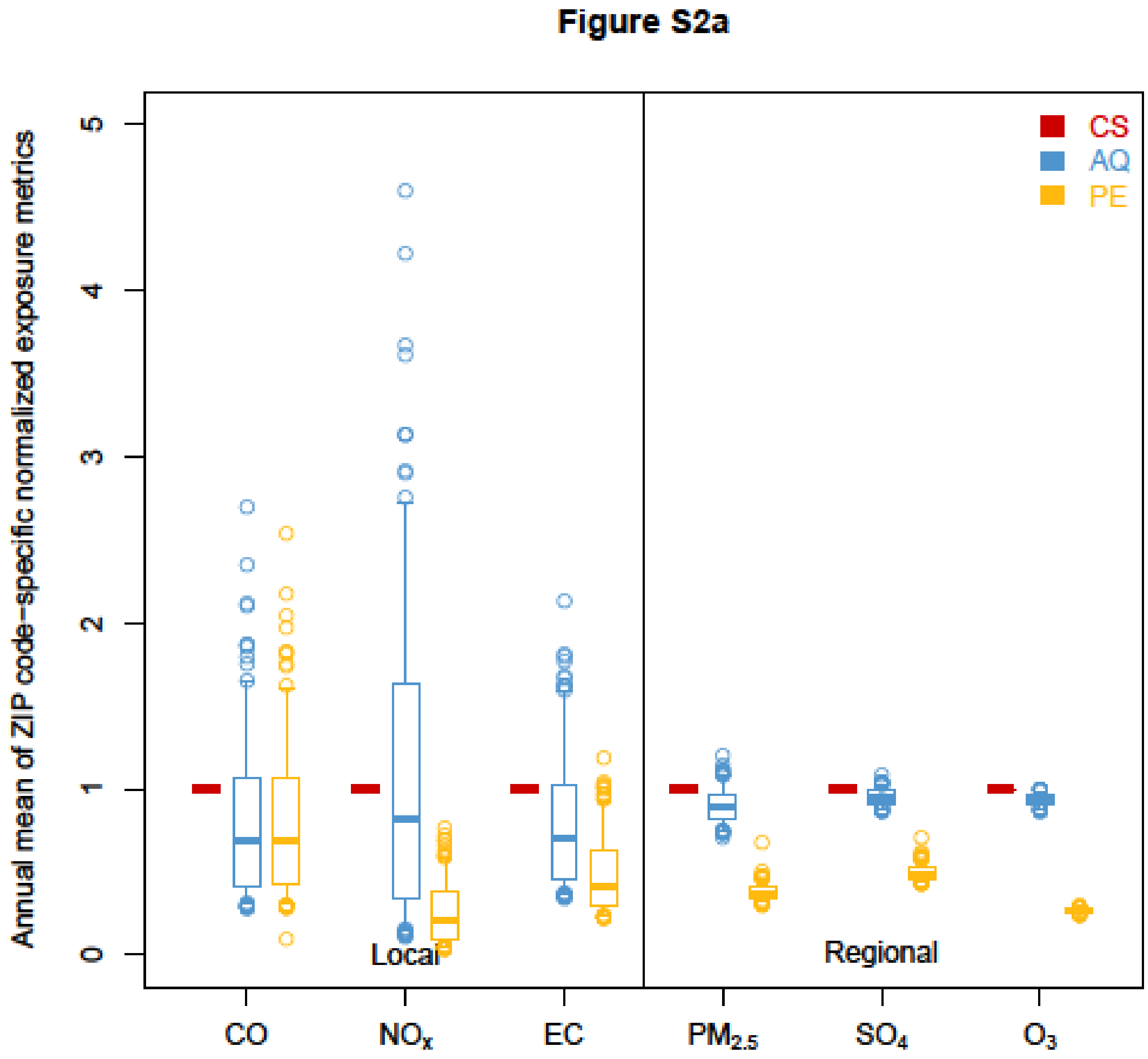

Figure S2a. Normalized exposure estimates from three methods: full extent of data and outliers (zoomed out version of Figure 1a; n=193 for each box). The bottom and top of the box represent 25th and 75th percentiles, the band near the middle of the box is the median, and the ends of the whiskers are the 5th and 95th percentiles.

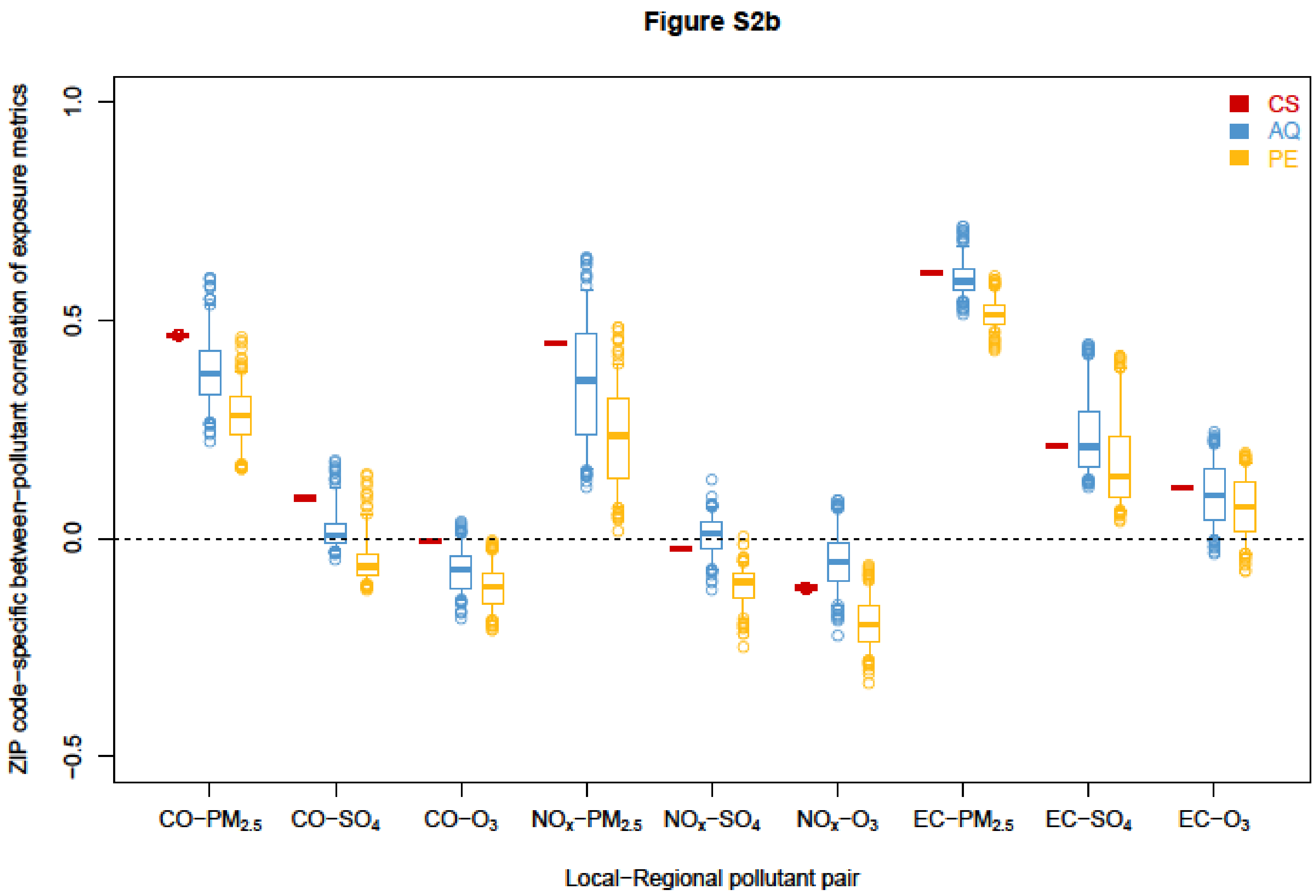

Figure S2b. Between-pollutant correlations of exposure for local-regional pollutant pairs; n=193 for each box. The bottom and top of the box represent 25th and 75th percentiles, the band near the middle of the box is the median, and the ends of the whiskers are the 5th and 95th percentiles.

Figure S3a

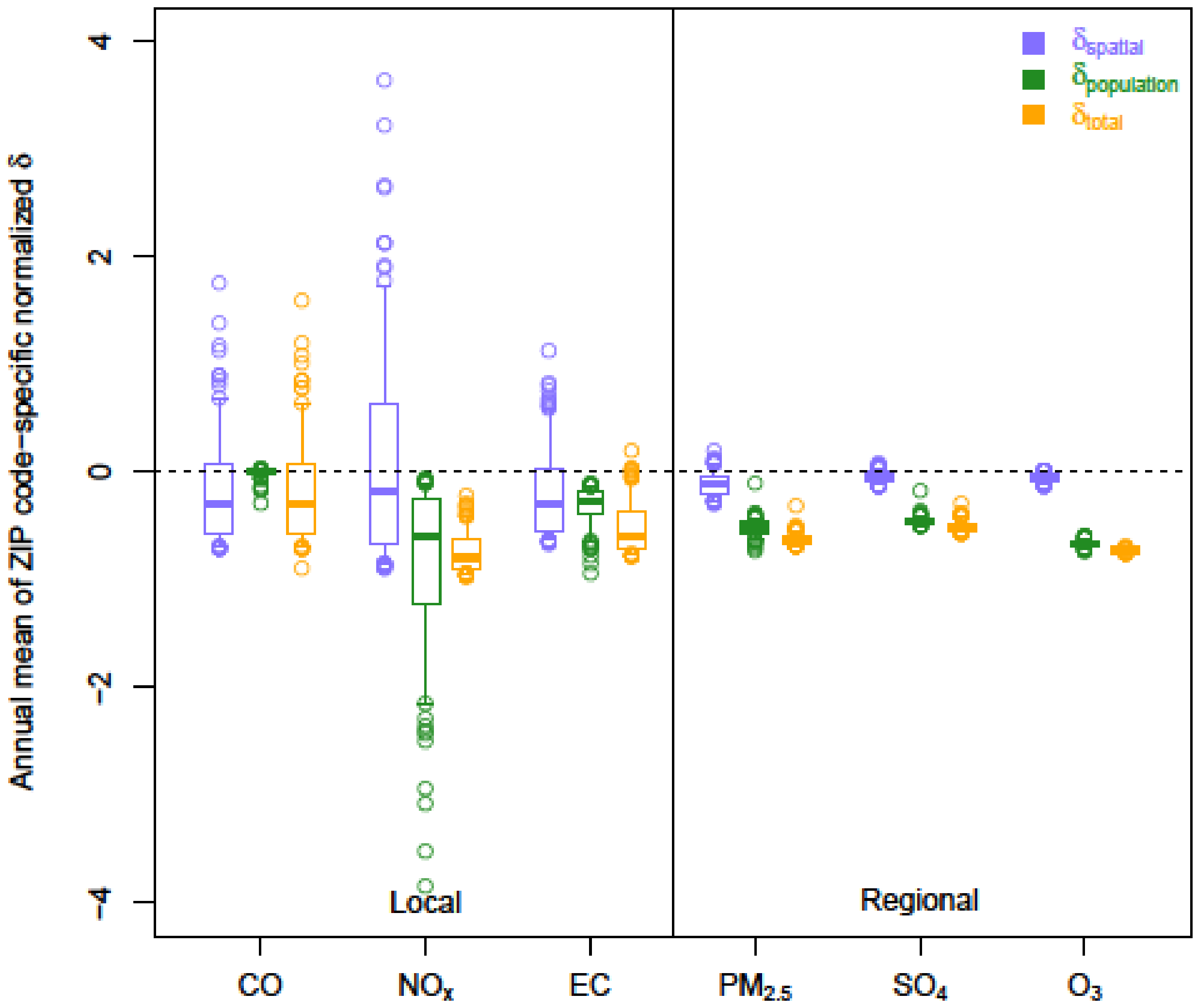

Figure S3a. Normalized exposure error: full extent of data and outliers (zoomed out version of Figure 2a; n=193 for each box). The bottom and top of the box represent 25th and 75th percentiles, the band near the middle of the box is the median, and the ends of the whiskers are the 5th and 95th percentiles.

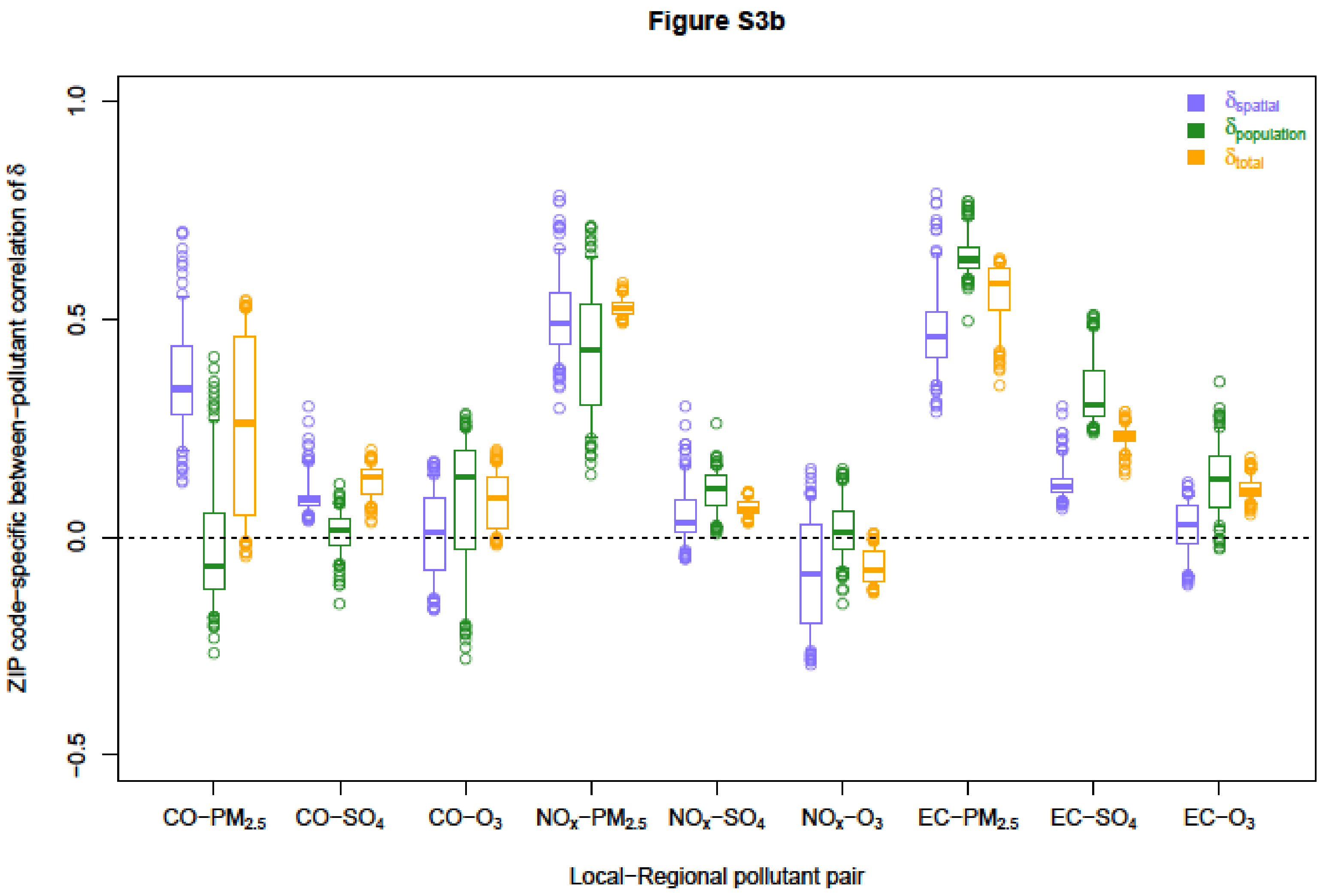

Figure S3b. Between-pollutant correlations of exposure error for local-regional pollutant pairs; n=193 for each box. The bottom and top of the box represent 25th and 75th percentiles, the band near the middle of the box is the median, and the ends of the whiskers are the 5th and 95th percentiles.

Figure S4

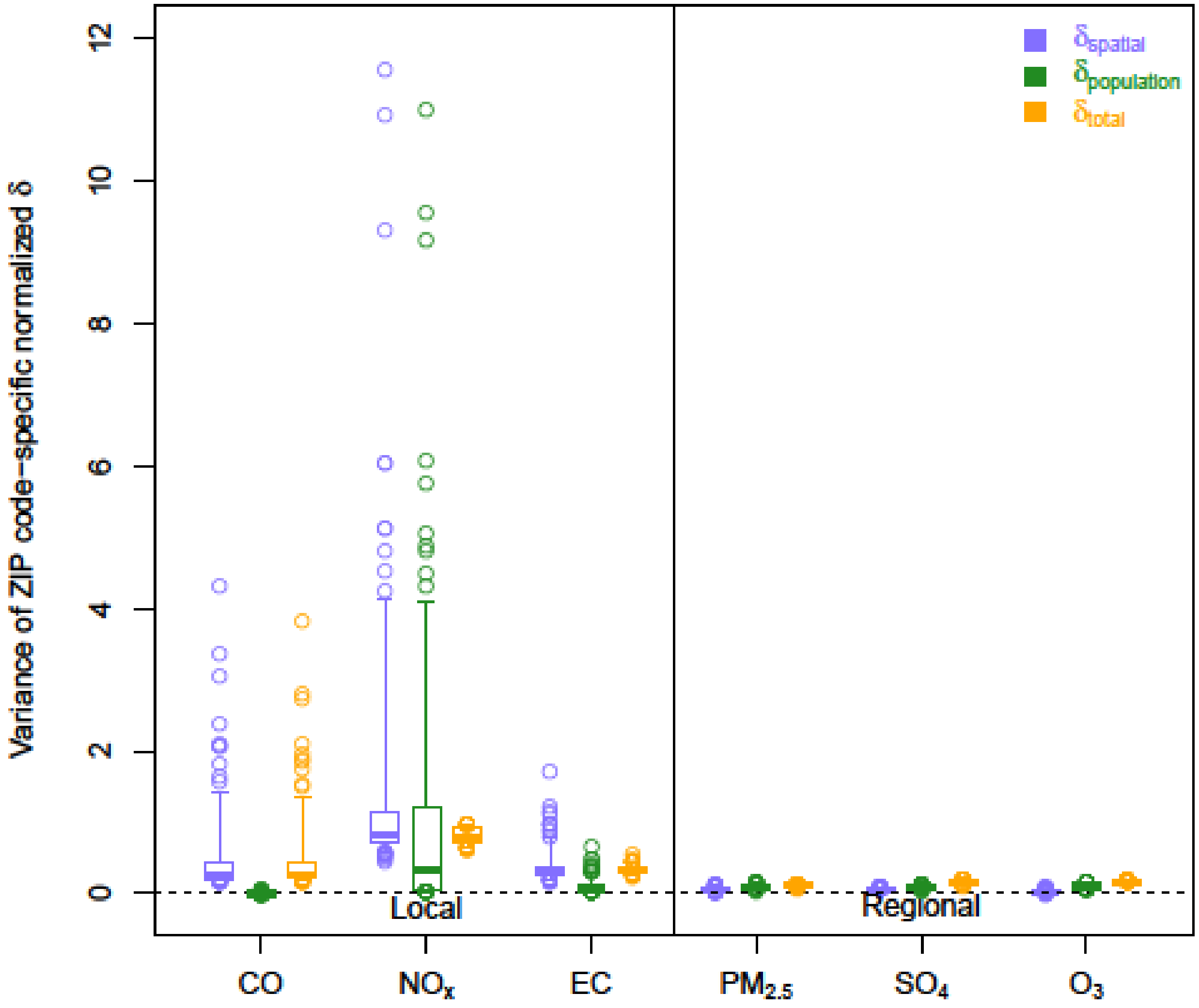

Figure S4. Variance of normalized exposure error: full extent of data and outliers (zoomed out version of Figure 3a; n=193 for each box). The bottom and top of the box represent 25th and 75th percentiles, the band near the middle of the box is the median, and the ends of the whiskers are the 5th and 95th percentiles.

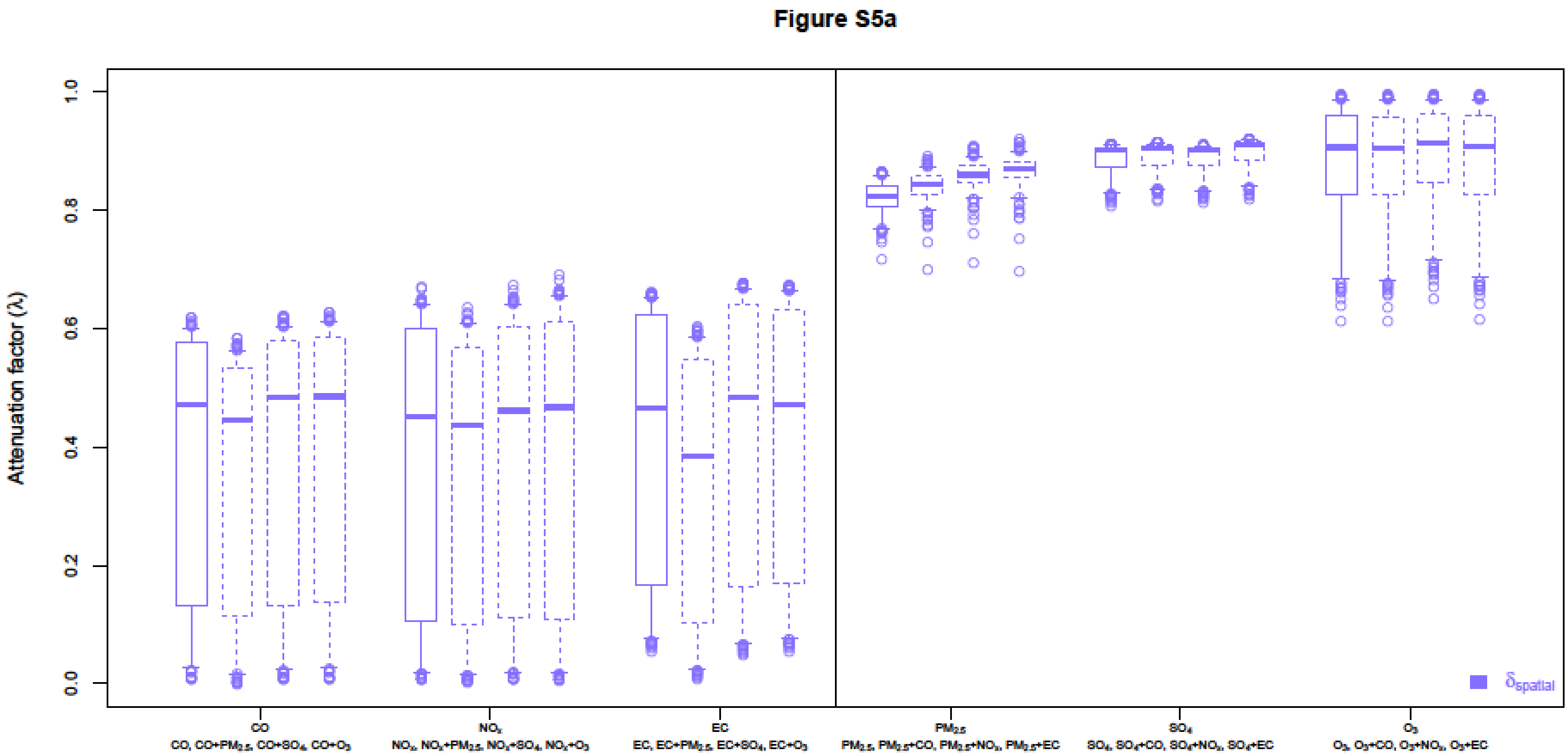

Figure S5a. Attenuation of model coefficients in a classical error, single pollutant framework, and in bi-pollutant models, assuming one pollutant has an effect, and one pollutant has no effect; $\delta_{spatial}$ for local-regional pollutant pairs; n=193 for each box. The bottom and top of the box represent 25th and 75th percentiles, the band near the middle of the box is the median, and the ends of the whiskers are the 5th and 95th percentiles.

Figure S5b

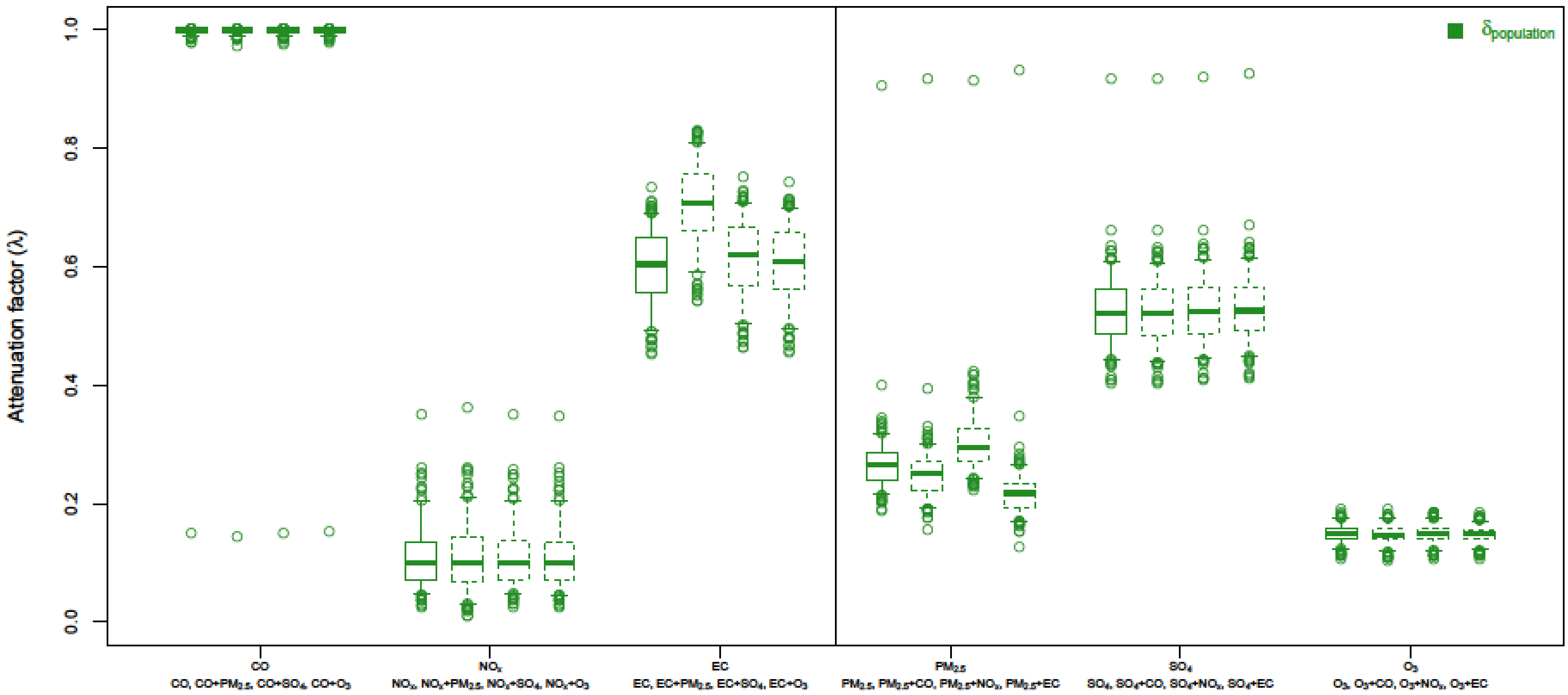

Figure S5b. Attenuation of model coefficients in a classical error, single pollutant framework, and in bi-pollutant models, assuming one pollutant has an effect, and one pollutant has no effect; $\delta_{population}$ for local-regional pollutant pairs; n=193 for each box. The bottom and top of the box represent 25th and 75th percentiles, the band near the middle of the box is the median, and the ends of the whiskers are the 5th and 95th percentiles.

Figure S5c

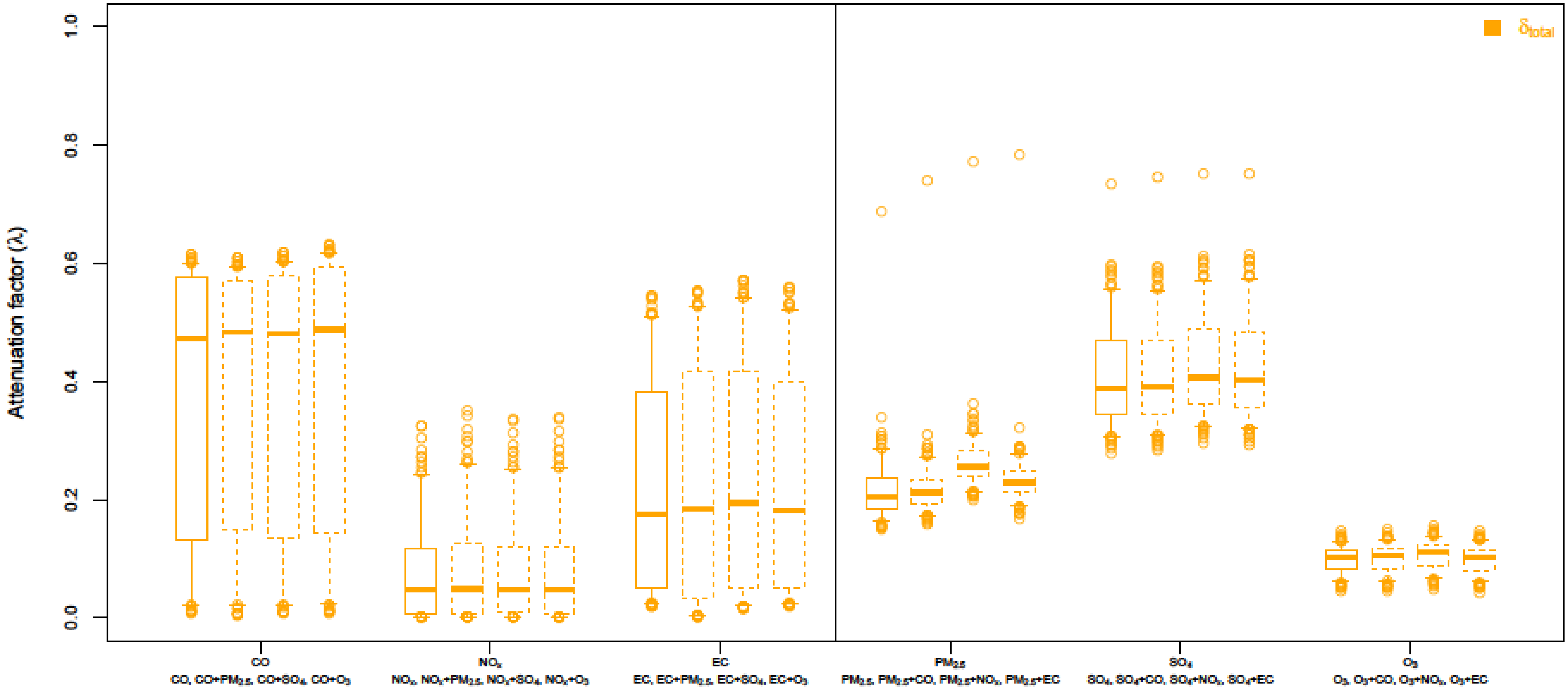

Figure S5c. Attenuation of model coefficients in a classical error, single pollutant framework, and in bi-pollutant models, assuming one pollutant has an effect, and one pollutant has no effect; δ_{total} for local-regional pollutant pairs; n=193 for each box. The bottom and top of the box represent 25th and 75th percentiles, the band near the middle of the box is the median, and the ends of the whiskers are the 5th and 95th percentiles.
